# Trophic strategies explain the ocean niches of small eukaryotic phytoplankton

**DOI:** 10.1098/rspb.2022.2021

**Published:** 2023-01-25

**Authors:** Kyle F. Edwards, Qian Li, Kelsey A. McBeain, Christopher R. Schvarcz, Grieg F. Steward

**Affiliations:** ^1^ Department of Oceanography, School of Ocean and Earth Science and Technology (SOEST), University of Hawaiʻi at Mānoa, Honolulu, HI 96822, USA; ^2^ Daniel K. Inouye Center for Microbial Oceanography: Research and Education, School of Ocean and Earth Science and Technology (SOEST), University of Hawaiʻi at Mānoa, Honolulu, HI 96822, USA; ^3^ School of Oceanography, Shanghai Jiao Tong University, 1954 Huashan Rd, Shanghai Shi, Xuhui Qu 200240, China

**Keywords:** mixotrophy, grazing, traits, tradeoffs, community structure, *Prochlorococcus*

## Abstract

A large fraction of marine primary production is performed by diverse small protists, and many of these phytoplankton are phagotrophic mixotrophs that vary widely in their capacity to consume bacterial prey. Prior analyses suggest that mixotrophic protists as a group vary in importance across ocean environments, but the mechanisms leading to broad functional diversity among mixotrophs, and the biogeochemical consequences of this, are less clear. Here we use isolates from seven major taxa to demonstrate a tradeoff between phototrophic performance (growth in the absence of prey) and phagotrophic performance (clearance rate when consuming *Prochlorococcus*). We then show that trophic strategy along the autotrophy-mixotrophy spectrum correlates strongly with global niche differences, across depths and across gradients of stratification and chlorophyll *a*. A model of competition shows that community shifts can be explained by greater fitness of faster-grazing mixotrophs when nutrients are scarce and light is plentiful. Our results illustrate how basic physiological constraints and principles of resource competition can organize complexity in the surface ocean ecosystem.

## 1. Introduction

Photosynthesis is the foundation of Earth's ecosystems and half of the daily primary production on the planet occurs in the surface ocean [1]. Most of this marine primary production is carried out by single-celled phytoplankton from a broad spectrum of ancient evolutionary lineages. Many eukaryotic members of the phytoplankton live a dual lifestyle as phagotrophic mixotrophs, meaning they photosynthesize but also consume prey [2]. The functional capabilities of many phytoplankton taxa are not known in detail, and the conditions that select for autotrophic versus mixotrophic strategies are not well established. This is particularly true for eukaryotic phytoplankton smaller than approximately 5 µm, which rival cyanobacteria as key photosynthesizers in the extensive oligotrophic ocean [3–5], while also being major predators of bacteria [6–8]. These organisms come from many deeply diverging lineages and encompass a rich diversity that exceeds that of smaller picocyanobacteria or larger microphytoplankton. Molecular surveys show co-occurrence of many higher taxa, such as haptophytes, chlorophytes, chrysophytes, dictyochophytes, pelagophytes, cryptophytes, bolidophytes, chlorarachniophytes, dinoflagellates and diatoms [9–11]. Habitat differences among clades imply functional diversity [12–15] but understanding how the traits that could drive these differences vary across clades is hampered by a paucity of experiments on isolates [3,16], especially those cultivated from the open ocean. Trophic strategy is probably an important axis of divergence: most taxa include phagotrophic mixotrophs that make their own chloroplasts [6,17], and feeding rates vary across clades [17], but groups such as non-flagellated prasinophytes likely cannot ingest prey, and this may be true of some flagellates as well [18–20]. An analysis of metatranscriptomes in the North Pacific, guided by machine learning of gene families associated with different trophic modes, predicted substantial variation in trophic mode across protist species, and shifts both within species and across communities along a latitudinal gradient [21]. Thus there is likely a spectrum of co-occurring trophic strategies, ranging from strictly autotrophic to largely heterotrophic phytoplankton, but quantitative data on how key functions vary across co-occurring species, and across major community gradients, is scarce. Models predict that mixotrophy in an ecosystem should generally increase primary production, trophic transfer efficiency and carbon export, while decreasing nutrient remineralization [22,23]. Climate change is expected to expand the nutrient-depleted oligotrophic gyres, potentially reducing global productivity [24], but mixotrophy has the potential to mitigate this outcome. Therefore a better understanding of the drivers of mixotrophy across ocean habitats, and the traits of the dominant taxa, may have broad consequences.

The relative fitness conferred by a given trophic strategy will depend on resource competition and the tradeoffs that constrain trait evolution. Photosynthesizers that consume prey benefit from multiple sources of nutrients and energy, but also experience competition from multiple directions, competing with specialized autotrophs for dissolved nutrients and light, and with specialized heterotrophs for prey [25]. Mixotrophs must allocate biomass and energy among a greater number of functions than specialists, which should reduce mass-specific photosynthesis compared to autotrophs. Likewise, they may ingest prey more slowly than heterotrophs if they invest less in phagotrophy, and the operation of multiple trophic modes could increase respiratory demand [26,27]. However, quantification of such tradeoffs is limited, and trait comparisons have focused mostly on larger coastal dinoflagellates [28] and some chrysophytes [26,29]. For example, mixotrophic dinoflagellates tend to have lower maximal ingestion rates than similar-sized heterotrophic dinoflagellates [28], and they also grow more slowly than similar-sized autotrophic diatoms if dissolved nutrients and light are the only resources [30]. It is unknown whether similar tradeoffs constrain the broader array of mixotrophic phytoplankton in open-ocean ecosystems.

Tradeoffs among trophic strategies should cause community structure to vary in predictable ways across environmental gradients. Compared to similar-sized autotrophs, phagotrophic mixotrophs should have a competitive advantage when dissolved nutrients are scarce relative to nutrients available in prey, or when light energy is limited relative to the chemical energy that can be derived from prey [25]. At the same time, mixotrophs should do worse than strict heterotrophic predators under low light, because photosynthesis by mixotrophs is too low to compensate for their lower ingestion rates. Under high light, however, mixotrophs are expected to outperform the heterotrophs, because the energy subsidy from photosynthesis should allow them to suppress prey to densities too low to sustain heterotrophs [31]. Therefore, the fitness of a mixotrophic strategy depends on relative supply of different resources as well as key tradeoffs, which combine to determine the net outcome of competition with multiple specialists.

Models using reasonable assumptions have found that well-lit environments with low nutrient supply may be most favourable for mixotrophs [32,33], but critical physiological parameters remain poorly constrained. In a synthesis of *in situ* experiments, lower latitude environments with greater irradiance showed increasing abundance of mixotrophs relative to specialists, and mixotrophs also increased relative to heterotrophs (but not autotrophs) in nutrient-rich coastal environments, patterns which were mostly consistent with model predictions [33]. This prior analysis considered mixotrophs and autotrophs as aggregates, but the extensive diversity within these groups raises the question of whether niche differences across taxa can be explained by trophic strategies, and whether quantifying mixotrophs in aggregate obscures important functional variation. If mixotrophs vary in their allocation of resources to different functions then the most successful strategy may vary continuously across gradients of light, nutrients, and prey [27]. Selection for different strategies across gradients, combined with physical mixing of plankton communities, may help explain the high local diversity of small phytoplankton [34], while also leading to gradients in ecosystem function.

In this study we combine three approaches to characterize functional diversity and community structure in a diverse guild of small open-ocean phytoplankton. First we assess tradeoffs between phototrophic performance (growth in the absence of prey) and phagotrophic performance (clearance rate when consuming *Prochlorococcus*), using a suite of 11 isolates representing a broad range of taxa and ecophysiologies. We then use clearance rates from these isolates and others, combined with Tara Oceans metabarcode survey data, to ask whether the capacity to consume prey varies across environmental gradients in phytoplankton communities. In both of these analyses, we use both mixotrophic and autotrophic taxa, which allows us to consider tradeoffs and niche differences within the mixotroph functional group, while also asking whether diversity among the phytoplankton as whole is consistent with a phototrophy-phagotropy spectrum. Finally, we use a model constrained by experimentally measured tradeoffs to analyse the mechanisms that could explain observed community gradients.

## 2. Functional diversity and tradeoffs

**Figure 1.**
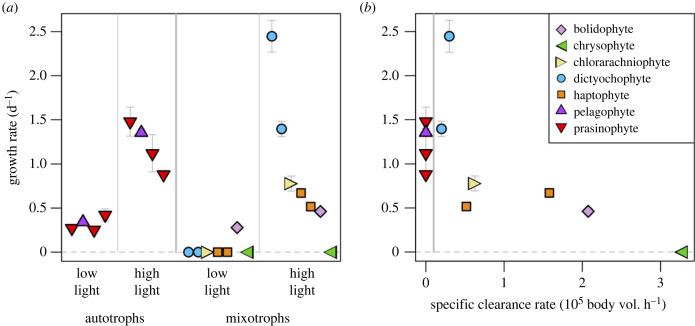
(*a*) Growth of 11 phytoplankton strains under phototrophic conditions (K medium, no added prey) when exposed to ‘high light’ (100 µ mol photons m^−2^ s^−1^) or ‘low light’ (10 µ mol photons m^−2^ s^−1^). Strains exhibiting no growth are given zeros. Strains are divided into autotrophs and mixotrophs, and those exhibiting no growth are given zeros. Error bars are ± 1 standard error of the mean; bars not visible are smaller than the point. (*b*) Growth rate under high light versus specific clearance rate when fed *Prochlorococcus*. Strains exhibiting no ability to grow when fed prey are given zeros. Strain names and growth data are listed in electronic supplementary material, table S1; clearance rates taken from [17].

To characterize how phototrophic and phagotrophic abilities covary across species we used eleven strains of less than 5 µm diameter eukaryotes isolated from the North Pacific Subtropical Gyre, representing eight classes that are widespread in the open ocean (electronic supplementary material, table S1 and figure S1). Capacity for phagotrophic mixotrophy was assayed as the ability to grow with *Prochlorococcus* prey (10^6^ ml^−1^) as the only added and significant source of nitrogen, under illuminated conditions (100 µ mol photons m^−2^ s^−1^). These conditions were previously shown to induce ingestion and phagotrophic growth in diverse isolates from this location [17]. In the current experiment four strains did not exhibit phagotrophic growth—two non-flagellated prasinophytes (*Ostreococcus*, *Chloropicon*), one flagellated prasinophyte (*Micromonas*) and one flagellated pelagophyte (*Pelagomonas*) (electronic supplementary material, figure S2). For simplicity, we will refer to these four strains functionally as ‘autotrophs’, while acknowledging the possibility that they ingest prey at very low rates that do not support growth, or could grow phagotrophically (or osmotrophically) on another food source. The remaining seven strains can grow phagotrophically (electronic supplementary material, figure S2) [17], and the rates at which they ingest *Prochlorococcus* were previously reported [17]. To characterize how phagotrophic capacity is related to phototrophy we measured growth of all strains under phototrophic conditions (addition of dissolved nutrients via K medium, but not prey) at a ‘high’ irradiance (100 µ mol photons m^−2^ s^−1^) that is the typical optimal irradiance for phytoplankton growth [30] and a ‘low’ irradiance (10 µ mol photons m^−2^ s^−1^) that is approximately 1% of surface PAR at the location from which these strains were isolated [35]. Under low irradiance, the autotrophic strains grew at rates of 0.25–0.42 d^−1^, while six of the seven mixotrophic strains failed to grow under these conditions (figure 1*a*). Under high irradiance, all strains could grow phototrophically except the chrysophyte (figure 1*a*). The high irradiance growth rates of autotrophs and mixotrophs overlap, although two strains of *Florenciella* were the only mixotrophs to grow faster than the slowest-growing autotrophs. *Florenciella* exhibits relatively low specific clearance rates (clearance rate normalized by predator biovolume), and across the mixotrophs the faster grazers tend to grow more slowly under high irradiance (figure 1*b*; Spearman *ρ* = –0.85, *p* = 0.024). A similar correlation is present when analysing the autotrophs and mixotrophs together (*ρ* = −0.74, *p* = 0.009).

In sum, these results imply that six of the mixotroph strains maintain the ability to grow photoautotrophically, but that greater grazing capacity is associated with a decline in phototrophic performance, which could be caused by reduced investment in photosynthetic machinery and/or greater respiratory demand. The cost of mixotrophy appears to be particularly high for phototrophic performance under light limitation because only one mixotroph could grow in this treatment. The chrysophyte, which has the fastest specific maximum clearance rate of any cultivated flagellate [17], may be an obligate mixotroph, as it did not grow when illuminated without added prey.

## 3. Relationship between trophic strategies and ocean niches

We next asked whether the trophic strategies of phytoplankton can explain their niches in the ocean. We combined (1) our assays of autotroph/mixotroph status; (2) our previously reported measurements of *Prochlorococcus* clearance rates for 29 mixotroph isolates (electronic supplementary material, table S2), 7 of which were also included in the phototrophic growth comparisons (figure 1); and (3) an analysis of environmental distributions using Tara Oceans metabarcoding data for pico/nanoeukaryotes (size fraction 0.8–5 µm) at 39 ocean stations. The Tara Oceans stations are primarily open ocean sites, with oligotrophic or mesotrophic characteristics (median surface total Chl *a*: 0.16 µg l^−1^; range: 0.011–0.63 µg l^−1^, electronic supplementary material, figure S3). Therefore, these samples represent the ocean environments in which small phytoplankton are most important, and our isolates were matched to metabarcode-based operational taxonomic units (OTUs) (Methods). On average the 13 OTUs studied here account for 31% of all metabarcode reads from non-dinoflagellate phytoplankton in this size fraction (Methods).

**Figure 2.**
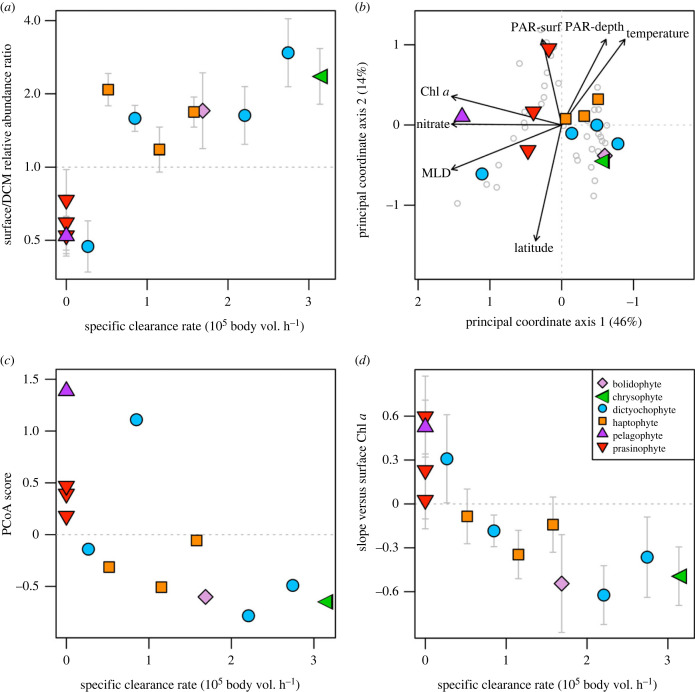
Environmental niches of 13 phytoplankton OTUs compared to grazing ability of corresponding isolates. (*a*) The *y*-axis is the ratio of OTU relative abundance in surface versus deep chlorophyll maximum (DCM) samples, derived from a GLMM fit to Tara Oceans metabarcodes in the 0.8–5 µm size fraction. The *x*-axis is grazing ability, quantified as specific clearance rate when fed *Prochlorococcus*, and strains exhibiting no ability to grow when fed prey are given zeros. (*b*) The first two axes from a principal coordinate analysis (PCoA) of OTU composition across surface samples. Vectors represent the correlations between environmental variables and the axes. PAR-surf = photosynthetically active radiation at the surface, PAR-depth = PAR at the sample depth, Latitude = absolute latitude. (*c*) OTU position along the first PCoA axis compared to grazing ability. (*d*) The *y*-axis is the slope of OTU relative abundance versus chlorophyll *a* concentration in surface samples, derived from a GLMM fit to Tara Oceans metabarcodes in the 0.8–5 µm size fraction. The *x*-axis is grazing ability as described for (*a*). Isolate/OTU names and clearance rate data are listed in electronic supplementary material, table S2. In panels (*a*), (*c*), and (*d*) the two dictyochophytes with specific clearance rate < 1 are *Florenciella* OTUs.

All mixotroph OTUs except one (*Florenciella* sp.) had greater relative abundance in surface samples than deep chlorophyll maximum (DCM) samples, while all autotroph OTUs had greater relative abundance in DCM samples (electronic supplementary material, figure S4). Furthermore, the surface:DCM relative abundance ratio is correlated with grazing ability (i.e. specific clearance rate when fed *Prochlorococcus*), such that better grazers have shallower distributions (figure 2*a*). The statistical relationship between grazing ability and depth niche is clearest when autotrophs and mixotrophs are both included—the 95% credible interval for the effect of clearance rate on the depth ratio is [0.18, 0.71], and *R*^2^ = 0.55 for the relationship between these two variables. However, the trend remains when only mixotrophs are considered [95% CI − 0.03, 0.72; *R*^2^ = 0.38], indicating that depth differences among mixotrophs correlate with their relative grazing abilities.

Much of the variation in OTU composition across surface samples can be explained by a single principle coordinate axis (46%; figure 2*b*). This axis is strongly positively correlated with Chl *a*, nitrate and mixed layer depth, and moderately negatively correlated with temperature and photosynthetically active radiation (PAR) at the sample depth (figure 2*b*). Therefore, this axis likely represents community structure driven by stratification, with less stratified waters having deeper mixed layers, greater nutrient supply and Chl *a*, and PAR diminished by greater pigment concentration. The position of OTUs along this axis is correlated with grazing ability (*r* = −0.67, *p* = 0.011; figure 2*c*), with autotrophs and slower-grazing mixotrophs more abundant under less stratified conditions. There is also a nonsignificant trend when only considering the nine mixotrophs (*r* = −0.5, *p* = 0.17). A similar but stronger pattern is found when considering niche differences across Chl *a* gradients (electronic supplementary material, figure S5). The four autotrophs and one mixotroph (*Florenciella* sp.) increase in relative abundance as Chl *a* increases, while the other mixotrophs decline, and better grazers show a steeper decline with increasing Chl *a* (figure 2*d*). The statistical relationship between grazing ability and Chl *a* niche is clearest when autotrophs and mixotrophs are both included [95% CI − 0.39, − 0.14; *R*^2^ = 0.9], but remains when only mixotrophs are considered [95% CI − 0.37, −0.06; *R*^2^ = 0.8]. Increasing the phylogenetic scale of these analyses, such that grazing abilities of isolates are matched to average niches of their respective families or orders, yield similar patterns, implying that the trait–niche relationships of OTUs are reflective of broader phylogenetic structure in these communities (electronic supplementary material, figure S6). Furthermore, when focusing on the portion of the phytoplankton community composed of these broader taxa, the community average grazing ability increases by a factor of approximately 4 across the stratification axis, and by a factor of approximately 2.5 between DCM and surface samples (electronic supplementary material, figure S7).

**Figure 3.**
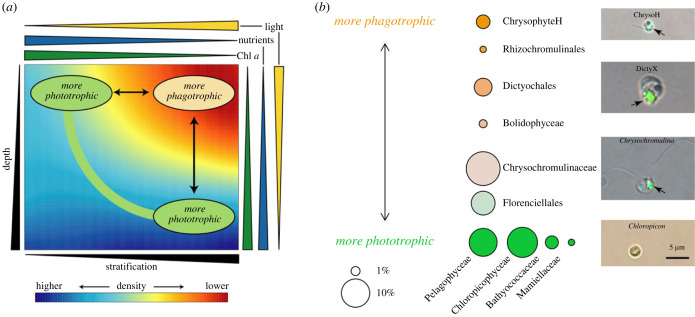
(*a*) Diagram of how depth and stratification gradients lead to parallel changes in phytoplankton community trait structure. Eukaryotic phytoplankton become more phagotrophic at shallower depths and in more stratified surface waters. Greater stratification decreases nutrient supply across the nutricline, and increases light intensity by reducing mixed layer depth and light-absorbing phytoplankton pigments. Moving from deeper to shallower depths is also associated with less nutrients and more light, and lower Chl *a*. (*b*) Diagram of the hypothesized spectrum of phytoplankton trophic strategies, based on traits of isolates and niches of OTUs and broader taxa (figures 1 and 2, electronic supplementary material, figures S6 and S7). Bubble area is proportional to mean relative abundance in surface Tara Oceans samples, within the 0.8–5 µm size fraction of phytoplankton (as defined in Methods). Images show example isolates from common taxa, arrows point to fluorescent beads ingested by mixotrophs as described in Li *et al*. [36]. In panel (*a*), moving across depth or stratification gradients leads to parallel shifts in relative abundance of taxa across the spectrum in panel (*b*).

The trait–niche relationships in figure 2 indicate that there are parallel changes in community structure when transitioning from deeper to shallower depths in the euphotic zone, and when transitioning from less stratified, high Chl *a* locations to more stratified, lower Chl *a* locations. Both of these gradients are associated with shifts from autotrophs and slower-grazing mixotrophs to faster grazing mixotrophs (figure 3); they are also associated with concomitant changes in the availability of nutrients and light, resources known to affect the relative fitness of different trophic strategies.

## 4. Trait-based model of trophic strategies across environmental gradients

**Figure 4.**
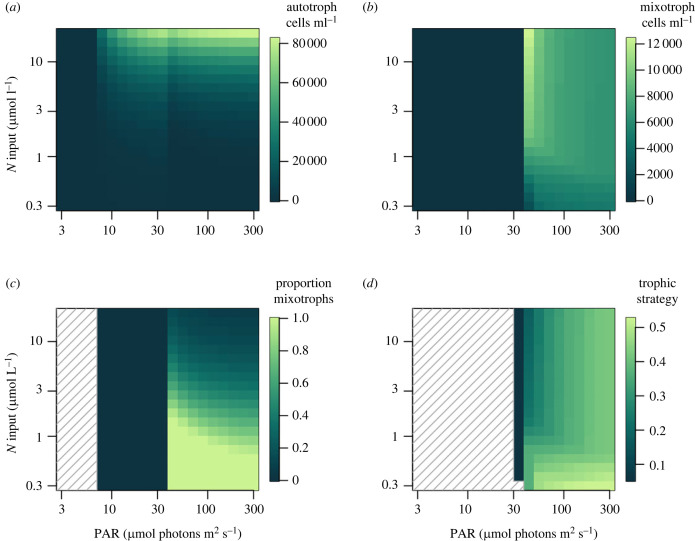
Modelled trophic strategies versus gradients of nitrogen input and irradiance, under the best-fit value of the tradeoff parameter (*φ* = 1.8). (*a*) Concentration of autotrophs. (*b*) Concentration of mixotrophs. (*c*) Mixotrophs as a proportion of all phytoplankton. (*d*) Trophic strategy of the persisting mixotroph population, where strategy is the parameter *x* (see Methods) which ranges from 0 (autotroph) to 1 (heterotroph). Regions with cross-hatching denote where phytoplankton did not persist (panel (*c*)) or mixotrophs did not persist (panel (*d*)). For brevity the heterotroph population is omitted (see electronic supplementary material, figures S9 and 10).

Because multiple environmental factors covary across gradients such as depth and stratification we used a model to consider whether the observed community patterns could be driven by shifts in nutrient supply, irradiance or both. We performed new analyses of a previously published model [33], where a spectrum of populations with different traits compete for dissolved nutrients and bacterial prey at a defined irradiance. The previous analyses focused on drivers of mixotrophs as an aggregate group, under hypothesized tradeoff assumptions, while the new analyses focus on trait variation among the mixotrophs, and use our experimental data to constrain the tradeoff where populations that ingest prey faster possess lower rates of photosynthesis (electronic supplementary material, figure S8). The data do not tightly constrain the model parameters but provide a range of plausible values to consider (electronic supplementary material, table S3). A notable result is that the tradeoff parameter *φ* has a best estimate of 1.8, with 95% confidence interval [0.98, 3.3]. This indicates the phototrophy–phagotrophy tradeoff may be fairly strong, because *φ* > 1 means an increase in one function causes a disproportionate decline in the other; for *φ* = 1.8 a mixotroph with 50% of the photosynthetic capacity of an autotroph has only 29% of the ingestion capacity of a heterotroph. The model predicts that an increase in nutrient supply causes autotrophs to increase relative to mixotrophs and at the same time mixotrophic strategies that invest more in phototrophy increase relative to strategies that invest less in phototrophy (i.e. the mean trophic strategy parameter declines; figure 4). The effect of irradiance is somewhat sensitive to tradeoff strength—under the best fit value of the tradeoff parameter, autotrophs are most competitive at the lowest irradiances, although there is a modest increase in mixotroph frequency from high to intermediate irradiances, while within the mixotrophs relatively phototrophic strategies increase as irradiance declines (figure 4). Under stronger tradeoff values the same qualitative patterns occur, but mixotrophs are restricted to lower nutrient inputs and higher irradiances (electronic supplementary material, figure S9). By contrast, under the weakest tradeoff values consistent with the data the mixotrophs can outcompete heterotrophs and autotrophs at lower irradiances, and mixotrophs become more phagotrophic under those conditions (electronic supplementary material, figure S10). By contrast to the irradiance gradient, the effect of the nutrient gradient is not qualitatively affected by tradeoff intensity.

In total, the model results suggest that strong relationships between grazing ability and environmental niches across multiple gradients (figures 2 and 3) may be driven by nutrient supply and potentially irradiance. Moving from shallow to deep within the euphotic zone is associated with increasing nutrient supply as well as declining irradiance. Likewise, nutrient supply increases and irradiance declines when moving from more stratified/low Chl *a* waters to less stratified/high Chl *a* waters. Nutrient supply across these gradients should favour relatively phagotrophic phytoplankton at shallower depths and in more stratified water columns (figure 4). The role of irradiance is more complex, but under relatively strong tradeoffs consistent with our data the greater irradiance at shallower depths and in more stratified water columns should also favour more phagotrophic phytoplankton (figure 4). It is noteworthy that gradients of the absolute abundance of mixotrophs can differ from their relative abundance as a proportion of the phytoplankton—e.g. greater concentrations of mixotrophs are predicted at higher nutrient concentrations, but autotrophs increase with nutrients at a faster rate. We have focused on nutrient and light axes to illustrate community outcomes, because these resources can vary greatly across ocean environments, but prey availability is implicitly important as well, as the ratio at which resources are supplied is the major driver of competitive outcomes [25,33,37]. Accordingly, an increase in prey availability with other resources held constant favours mixotrophs over autotrophs, and favours relatively phagotrophic strategies among the mixotrophs (results not shown).

## 5. Discussion

Understanding functional diversity in marine protists and its ecosystem consequences is challenging, because of high taxonomic diversity, sparse sampling, ecosystem complexity and the advantages and limitations of different methodologies. The approach taken here uses experiments with diverse isolates to test hypothesized tradeoffs and link trait differences to niche differences. Although these analyses include a subset of total phytoplankton diversity, even within this size range, a broad range of mixotrophic functional diversity is explicitly represented. One alternative approach to quantifying mixotrophy is fluorescent labelling of prey, to count the total abundance of mixotrophic nanoflagellates *in situ*, as well as co-occurring autotrophs and heterotrophs (e.g. [38,39]). This approach probably underestimates the true abundance and/or ingestion rates of mixotrophs [40], but similar general patterns emerge using different approaches. A prior synthetic analysis of labelled-prey experiments found two major patterns: mixotrophs tend to increase in abundance at lower latitudes, while autotrophs and heterotrophs show little latitudinal trend; and all three groups increase in abundance in productive coastal environments, but autotrophs and mixotrophs increase much more steeply than heterotrophs [33]. It was argued that these patterns may result from the relative supply of light, nutrients and prey along these gradients. Lower latitudes have greater incident irradiance and a tendency for lower nutrient supply. Greater irradiance makes mixotrophs more competitive against heterotrophs, while reduced nutrient supply makes mixotrophs more competitive against autotrophs, allowing mixotrophs to increase relative to both groups. The results of the current study appear consistent with these prior findings because greater stratification also leads to reduced nutrient supply and greater mixed-layer irradiance. Another recent study used protistan metatranscriptomes to investigate variation in trophic mode from the oligotrophic North Pacific subtropical gyre into the transition zone to the north [21]. In the small eukaryote size fraction (0.2–3 µm) they found high proportions of gene families associated with heterotrophy in the gyre, while gene families associated with mixotrophy and photoautotrophy increased towards the transition zone. It is not clear whether plastid-bearing protists expressing heterotrophy-associated gene families are wholly heterotrophic, or performing a relatively heterotrophic form of mixotrophy, such as photoheterotrophy [41]. Nonetheless, these results are also consistent with a general shift from more phagotrophic to more phototrophic strategies as nutrients increase and irradiance declines.

In the previous compilation of labelled prey experiments, the coastal-to-open ocean gradient was primarily associated with large increases in nutrient supply and Chl *a* in more coastal environments and a more modest increase in bacterial abundance. Increases in the nutrient:prey supply ratio are expected to favour mixotrophs more than heterotrophs, and autotrophs more than mixotrophs [33]. The fact that mixotrophs and autotrophs tended to increase in parallel was surprising. The apparent similarity in trends could result from insufficient statistical power to detect a difference in slopes or may reflect substantial increases in relatively autotrophic mixotrophs, such as *Florenciella*, in nutrient-rich environments. This genus is globally abundant [17], grazes relatively slowly [17] (figure 2), grows quickly on dissolved nutrients [36] (figure 1), and contains the one mixotroph OTU in our analysis that increases in relative abundance across a Chl *a* gradient (figure 2; electronic supplementary material, figures S4 and S5). This demonstrated variation in traits among mixotrophs, which is correlated with environmental gradients, implies that bulk measurements of mixotroph abundance may obscure significant shifts in community function. The population-level analysis used in the current study benefits from having multiple representatives of both autotrophs and mixotrophs, and as well as functional diversity within the mixotrophs. This provides a form of statistical replication when testing how the environment drives trophic strategies, and allows for insights into whether the mixotrophs themselves change in important ways across environments.

It is noteworthy that a single axis of phototrophy versus phagotrophy seems to capture much of the ecological variation among taxa from many deeply branching clades. This suggests that fundamental constraints on physiology may lead to ‘universal’ tradeoffs that underlie trait diversity, community structure, and ecosystem function. The current study assesses phototrophy/phagotrophy tradeoffs at a relatively high level: isolates that ingest prey more rapidly grow more slowly when prey are not available. It will be important to make detailed physiological comparisons across diverse mixotroph taxa to understand cellular mechanisms underlying this relationship. For example, quantification of proteins used for carbon fixation, and pigments and proteins used to harvest light, would reveal whether allocation to these functions drives phototrophic growth rate and covaries with ingestion rate across taxa. Assessing plasticity in photosynthesis and ingestion, and coupling between photosynthesis and respiration, would also help reveal whether taxa with different average strategies regulate mixotrophy in distinct ways. For example, studies by Wilken *et al*. [29] and Fischer *et al*. [42] both compared pairs of chrysophytes and found that some species are obligate mixotrophs that require light and prey for growth, while others are facultative mixotrophs that can grow in darkness with sufficient prey and survive in the light without prey. Obligate mixotrophs may invest more in light capture [42], and have tighter integration of autotrophic and heterotrophic metabolism [29], leading to greater efficiency and faster growth under stable stratified conditions, while a facultative strategy could be more advantageous in variable environments.

A fuller accounting of eukaryotic diversity will require isolation of additional common clades, as well as *in situ* methods for quantifying trait variation [20,21]. It would also be worthwhile to investigate additional sources of niche variation among the taxa studied here; for example, the mixotrophs in figure 2 vary from 2.3 to 4.7 µm in cell diameter, which could lead to differences in diet and/or predators. In addition, the relative fitness of mixotrophic strategies depends on competition with heterotrophs, which have not been examined in this study. Isolation of prevalent open-ocean heterotrophs, and comparison of their traits and niches to co-occurring mixotrophs, would further refine our understanding of the competitive interactions that structure plankton communities. Recent work has shown that mixotrophs that do not make their own chloroplasts have biogeographies that depend on the mode of chloroplast acquisition [43], and modelling shows that non-constitutive mixotrophs may have a different niche than the constitutive form [44]. Integrating the spectrum of constitutive and acquired mixotrophies into a unified analysis of competitive outcomes should provide a more complete understanding of trophic strategies among unicellular protists.

The observed patterns in community trait structure imply gradients in ecosystem functioning, such that phagotrophically supported primary production may be greatest at shallow depths of highly stratified waters, which is predicted to influence trophic transfer efficiency and nutrient cycling [22,23]. There are many potential drivers of stratification across the *Tara* Oceans survey locations, and one possibility is seasonal cycles that were at different stages at the different sampling stations. A seasonal component to the niche differences in figure 2*b–d* would mean that seasonal patterns among small eukaryotes are similar to those seen in larger microphytoplankton, where mixotrophic dinoflagellates become more abundant than autotrophic diatoms during stratified summer conditions [43], consistent with an optimality-based model of succession among trophic strategies [27]. Our results also suggest that climate change, which is increasing ocean stratification [44], is also making phytoplankton communities more phagotrophic. Finally, it is noteworthy that the correlation of grazing ability with a population's Chl *a* niche (figure 2*d*) provides a link to remote sensing. Phytoplankton community trophic strategy may be predictable at a global scale using remotely sensed Chl *a*, and this may also provide a route for phagotrophy to be better incorporated into models of primary production.

## 6. Methods

### (a) Strain isolation and cultivation

Isolation methods for most strains used in this study were described in Li *et al*. [17]. Briefly, the majority were isolated from the euphotic zone at Station ALOHA (22°45′ N, 158°00′ W) in 2019, enriched using Keller (K) medium with a 20-fold reduction in mineral nutrients and addition of *Prochlorococcus* (MIT9301) at approximately 5 × 10^6^ cells ml^−1^. Five strains were isolated from previous samples at the same location, enriched with full K medium or K medium without added nitrogen. Four strains used in the present study (*Ostreococcus*, *Chloropicon*, *Micromonas*, *Pelagomonas*) were not described in Li *et al*. [17]. These strains were isolated from the same location, enriched using full K medium. All strains were rendered unialgal but not axenic, maintained at 24°C in 0.2 µm-filtered and autoclaved ALOHA seawater, under a 12 : 12 light:dark cycle with irradiance approximately 70 µM photons m^−2^ s^−1^. Mixotrophs (as described in Li *et al*. [17]) were maintained in K medium without added nitrogen, amended with *Prochlorococcus* prey. The four strains not previously described were maintained in full K medium. Strain taxonomy was characterized with phylogenetic analysis of 18S rDNA as described in Li *et al*. [17].

### (b) Mixotrophy assays

Eleven strains were used to compare phototrophic growth abilities with the ability to grow when fed *Prochlorococcus*. Seven strains were previously shown to consume *Prochlorococcus* and grow when fed *Prochlorococcus* as the only added nitrogen source: a chrysophyte from environmental clade H (hereafter ChrysoH), a bolidophyte in the genus *Triparma*, two haptophytes in the genus *Chrysochromulina*, two dictyochophytes in the genus *Florenciella* and one undescribed chlorarachniophyte (hereafter ChloraX) [17]. These seven strains were selected from the larger set studied previously [17] in order to have representatives from diverse classes and to represent the full range of observed grazing abilities. Four strains were newly assayed for ability to grow when fed *Prochlorococcus*: three prasinophytes from the genera *Ostreococcus*, *Chloropicon* and *Micromonas*, and one pelagophyte in the genus *Pelagomonas*. Three previously assayed mixotrophs were included in the new assays (two *Chrysochromulina* and ChrysoH) as positive controls. Strains were inoculated into K medium without added nitrogen at approximately 10^3^ cells ml^−1^, at an irradiance of 100 µ mol photons m^−2^ s^−1^ (12 : 12 light : dark cycle), and monitored for eight days to allow consumption of residual nitrogen, at which point *Prochlorococcus* was added at approximately 10^6^ cells ml^−1^. In previous experiments using this *Prochlorococcus* strain under the same conditions, *Prochlorococcus* cells contained 15 fg N per cell. Cultures were monitored for eight more days for evidence of growth and compared to control cultures without added *Prochlorococcus*.

### (c) Phototrophic growth measurements

The eleven strains used in mixotrophy assays were also tested for phototrophic growth ability, i.e. the ability to grow using light and dissolved nutrients as resources. Cultures were inoculated at approximately 10^2^–10^3^ cells ml^−1^ into tissue culture flasks containing 20 ml *K* medium, at two irradiances (10 and 100 µ mol photons m^−2^ s^−1^), referred to as ‘low’ and ‘high’ light, respectively. Samples were taken every 1–3 days and incubated in a final volume of 0.5% glutaraldehyde for 15 min before flash freezing in liquid nitrogen and storage at −80°C, followed by counts with flow cytometry. All strains were acclimated by passaging through at least one batch culture in the experimental conditions, before collecting data to estimate growth rates. Some strains grew at high irradiance but consistently failed to grow at low irradiance after repeated inoculations, as noted in the main text. One strain (ChrysoH) was unable to grow phototrophically (i.e. without added prey), although it grew readily with added prey. For strains that grew, growth rate was estimated using at least two replicates in all cases, except one *Chrysochromulina* strain for which one high light growth rate was obtained.

Growth rates were estimated by fitting nonlinear growth models to cell concentrations over time. For cultures that exhibited a lag before exponential growth a growth model with a lag phase and carrying capacity was fit:

$$\ln(\;C(t))=\frac{\ln(K)}{1+\exp[(4\mu /\ln(\;K))(\lambda -t)+2]},$$

where *C*(*t*) is cell concentration at time *t*, *µ* is the exponential growth rate, *K* is carrying capacity (stationary density) and $\dot{\lambda }\;$ is the inflection point where growth rate equals µ [45]. For cultures that exhibited no lag a logistic growth model was fit:

$$C(t)=\frac{K}{1+\exp[\;-\mu (t-t_{m})]},$$

where *t*_m_ is the inflection point of the logistic curve and *C(t)*, *K* and µ have the same meaning. The models were fit by maximum likelihood with the R package bbmle [46]. In cases where cell concentration declined after reaching maximal abundance, the decline phase was omitted to allow the model to fit to the sigmoidal portion of the growth curve.

### (d) Tara Oceans OTUs

In order to compare traits of our isolates to the environmental niches of their populations, or closely related populations, we used the Tara Oceans eukaryotic plankton diversity dataset [47]. This dataset contains size-fractionated 18S-V9 rDNA metabarcodes from 40 stations in the sunlit ocean (http://taraoceans.sb-roscoff.fr/EukDiv/; electronic supplementary material, figure S3). Using 27 strains for which we previously measured clearance rates when consuming *Prochlorococcus* [17], plus four additional strains assayed in this study (*Ostreococcus*, *Chloropicon*, *Micromonas*, *Pelagomonas*), we matched strains to OTUs from the Tara Oceans dataset (electronic supplementary material, table S2). Near-full length 18S rDNA sequences of our isolates were compared to all OTU 18S-V9 rDNA reference sequences using nucleotide BLAST. In nearly all cases the OTU with the lowest E-score was frequent enough to analyse abundance patterns across samples (i.e. thousands of reads or more), and this OTU was chosen for further analysis. In some cases, there was a second OTU with an equivalent E-score but less than 10 reads, and this OTU was not used. In one case (DictyX) the OTUs with the two lowest E-scores had less than 10 reads, and the third-ranking OTU was chosen. In one case (*Chloropicon*) two abundant OTUs had the same E-score, and their reads were summed in each sample for further analysis (choosing one OTU produced similar results). The chlorarachniophyte strain ChloraX was not similar to any OTU abundant enough for further analysis. *Florenciella* strains were divided into two groups, one group that best matched a *Florenciella parvula* OTU and one that matched an OTU from an undescribed *Florenciella* species. In all cases, taxonomic annotation of the *Tara* Oceans OTUs was consistent with isolate taxonomy independently derived by phylogenetic analysis of isolate 18S rDNA and related sequences from GenBank and the PR^2^ database [48].

### (e) Statistical analyses

We asked whether the environmental niches of phytoplankton OTUs are correlated with their grazing ability. Grazing ability was quantified as body volume-specific clearance rate when fed approximately 10^6^ cells ml^−1^ *Prochlorococcus* [17,48], and this trait was used because we have measured it on a large number of isolates. As described previously, the clearance rates measured with these isolates approximate their maximum clearance rates, because prey concentrations were low enough to not saturate the ingestion rate [17]. Isolates determined to be autotrophic by our mixotrophy assays were given a grazing ability of zero. This yielded a total of 13 OTUs for which niches could be compared to grazing ability. Metabarcodes from the ‘pico/nano’ 0.8–5 µm size fraction were used, as all of our isolates are within this size class.

We took two approaches to test whether grazing ability correlates with niche differences. We used principal coordinate analysis to ordinate major axes of compositional variation among our focal OTUs. To interpret drivers of composition we then correlated the first principal coordinate axis with environmental variables: Chl *a* concentration (HPLC), photosynthetically active radiation (PAR) at the sea surface, PAR at the sample depth, nitrate concentration, sea surface temperature, mixed layer depth and absolute latitude. All variables were taken from ancillary *Tara* Oceans datasets [49–51]. Finally, the position of OTUs along the first axis was compared to their grazing ability.

We also asked whether grazing ability was correlated with OTU responses to specific environmental variables chosen *a priori*: depth (surface [3–7 m] versus deep chlorophyll maximum [DCM]), and Chl *a* concentration. When using Chl *a* as the predictor only surface samples were used, and one station was withheld because it had much higher Chl *a* concentration (5.5 µg l^−1^) than the other stations (0.011–0.63 µg l^−1^). Samples without Chl *a* data were excluded in this analysis. For depth and Chl *a* generalized linear mixed models (GLMMs) were fit, with OTU relative abundances modelled using the beta-binomial distribution with a logit link function:

$$\begin{array}{rl} \mathrm{logit}(\;p_{\mathrm{ij}}) & ={\mathrm{Int}}_{i}+{\mathrm{Sample}}_{j}+({\mathrm{CR}}_{i}\times \mathrm{CReff}+{\mathrm{slope}}_{i})\\ & \;\times {\mathrm{Env}}_{j},\;\mathrm{read}s_{ij}∼\mathrm{BetaBinom}(\;p_{ij},V_{i},N_{j}). \end{array}$$

Here *p*_ij_ is the probability that a metabarcode read in sample *j* is from OTU *i*, *Int*_i_ is an OTU-specific random intercept capturing variation in mean relative abundance across OTUs, *Sample*_j_ is a random effect capturing variation in mean relative abundance of all OTUs across samples, *CR*_i_ is specific clearance rate of OTU *i*, *CReff* is the effect of clearance rate on OTU responses to the environment, *slope*_i_ is a species-specific random slope capturing variation in environmental responses not attributable to CR, *Env*_j_ is the value of the environmental variable in sample *j*, *reads*_ij_ is number of reads of OTU *i* in sample *j*, *V*_i_ is an OTU-specific dispersion parameter and *N*_j_ is the total number of phytoplankton reads in sample *j*. In summary, this model quantifies whether the relationship between relative abundance and an environmental variable for an OTU is predicted by that OTU's clearance rate. The GLMM approach is appropriate because it models # of reads while accounting for variation in total reads, and allows for uncertainty in relative abundances and environmental relationships while quantifying CReff [52,53]. The assumption of logit-linear environmental responses was appropriate based on visual inspection of the data (electronic supplementary material, figure S5). To account for potential phylogenetic correlation in OTU environmental responses we also fit models with additional random effects for taxon (haptophyte/dictyochophyte/prasinophyte/chrysophyte/pelagophyte/bolidophyte), but in all cases variance of these effects was zero. Models were fit in R using the package brms, which implements bayesian regression models via the software Stan [54]. All niche analyses were performed with two datasets: the full set of 13 OTUs including autotrophs and mixotrophs, and the set of 9 mixotroph OTUs. This allowed us to test for drivers of diversity within the mixotrophs, and also test whether including autotrophs as endmembers of the trophic spectrum yields consistent results.

To define the total number of phytoplankton reads we summed the reads of known phytoplankton taxa (*Tara* Oceans ‘taxogroups’: Bacillariophyta, Bolidophyceae, Chlorarachnea, Chlorophyceae, Chrysophycea/Synurophyceae, Cryptophyta, Dictyochophyceae, Euglenida, Glaucocystophyta, Haptophyta, Mamiellophyceae, Other Archaeplastida, Other Chlorophyta, Pelagophyceae, Phaeophyceae, Pinguiophyceae, Prasino-Clade-7, Pyramimonadales, Raphidophyceae, Rhodophyta, Trebouxiophyceae). Dinoflagellates were excluded because of the difficulty in assigning phototrophic versus heterotrophic status to all taxa, and because nearly all dinoflagellate reads were from a single, poorly annotated OTU that was also highly abundant in larger size fractions. We also excluded a small number of taxa within the taxogroups listed above that are known to be heterotrophic. However, neither the exclusion of dinoflagellates nor the heterotrophs within majority-phytoplankton taxogroups qualitatively changes our results.

### (f) Trait-based model of trophic strategy competition

In this study we perform new analyses of a model similar to that described by Edwards [33]. The model describes population growth of single-celled protists where the potential limiting factors are dissolved nutrients, bacterial prey, and light. We assume that species can vary continuously in allocating resources to phototrophy or phagotrophy, which means that the model includes autotrophs, heterotrophs and a spectrum of mixotrophs between these. Although our empirical analyses only include autotrophs and mixotrophs, heterotrophs are included in the model because they probably compete with mixotrophs for prey, and therefore affect the conditions under which different mixotrophic strategies are successful. The phototrophy-phagotrophy spectrum is modelled as a tradeoff between rates of photosynthesis and nutrient uptake (phototrophic functions) and rates of ingestion (phagotrophic function). The strength of this tradeoff is modelled using a parameter, *φ*, which controls the curvature of the tradeoff and therefore its strength: *φ* > 1 penalizes mixotrophs for a generalized strategy, while *φ* < 1 rewards them [33]. We used the data from our phototrophic growth experiments on 11 isolates to estimate *φ*, as well as the other model parameters that determine the light-limited growth rate in the absence of prey. To ask how community structure varies across environmental gradients we initialized communities with a spectrum of trophic strategies and allowed them to compete for nutrients and prey until a stable community of one or more species emerged. A full description of the model and its analysis is given in the electronic supplementary material Modelling Methods.

## Data Availability

Growth rates reported in figure 1, and Genbank accession numbers for 18S rDNA sequences of the isolates, are provided in electronic supplementary material, table S1. Clearance rates used in figure 2, R code used to analyse Tara Oceans data, and R code for the trait-based model, are provided in a electronic supplementary material zip file. The data are provided in electronic supplementary material [64].
